# If Providing Best Care Means Being at the Cutting Edge of Research, Should It Be Implemented System-wide? Comment on "‘We’re Not Providing the Best Care If We Are Not on the Cutting Edge of Research’: A Research Impact Evaluation at a Regional Australian Hospital and Health Service"

**DOI:** 10.34172/ijhpm.2023.7718

**Published:** 2023-06-18

**Authors:** Stephen R. Hanney

**Affiliations:** Health Economics Research Group, Department of Health Sciences, Brunel University London, London, UK

**Keywords:** Research Capacity, Research Engagement, Research Impact, Research Translation, Health Research Systems, COVID-19

## Abstract

The COVID-19 pandemic intensified debates about the desirability of integrating health research systems into healthcare systems. An excellent evaluation undertaken prior to the pandemic examined a purposeful strategy to improve healthcare through an expansion in research capacity in the Townsville Hospital and Health Service (THHS), a regional service in northern Queensland. This comment puts that evaluation into a rapidly developing wider context, drawing on other work showing an association between research engagement in healthcare organisations and their improved healthcare. In most previous studies this impact arose as a by-product of the research activity. The Townsville scheme went further. But while the evaluation identified some progress and impacts, they were patchy, not system-wide. Recent pre-pandemic studies showed that going even further and integrating a health research system across a national healthcare system markedly improved healthcare, despite continuing challenges. The UK’s research experiences during COVID-19 are giving additional momentum to this approach globally.

 The benefits from having a health research system embedded throughout the healthcare system were illustrated by the rapid progress made in the early months of the COVID-19 pandemic by the Randomised Evaluation of COVID-19 Therapy (RECOVERY) trial in the United Kingdom. This trial was conducted by clinical researchers in all UK acute hospital trusts who recruited the first 1000 patients in 15 days, and within 100 days identified dexamethasone as the first effective therapy to reduce mortality.^1^ This dramatic success highlights the need for further studies that explore how research systems can be embedded into healthcare organisations, and the gains that might arise outside a pandemic.

## An Australian Initiative

 A recently published paper from Australia by Amy Brown and colleagues entitled *“We’re Not Providing the Best Care If We Are Not on the Cutting Edge of Research”: A Research Impact Evaluation at a Regional Australian Hospital and Health Service*^2^ reports one relevant initiative undertaken between 2008 and 2018. The paper evaluates a purposeful strategy designed to achieve impacts such as improved patient care through the development of research capacity across Townsville Hospital and Health Service (THHS), a regional service in northern Queensland that was not a traditional major academic research centre. The quote in the title of the paper is from one of the interviewees in the innovative realist evaluation.

 This paper is the second from the evaluation and focuses on impact: the first paper described the features and goals of the research investment.^3^ Each paper can be read as a separate analysis, but their different scope means that it is useful to read both. They both emphasise that it was the intention that research activity would explicitly create impacts by improving healthcare practice, policy, and outcomes across THHS. The first paper explicitly stated: *“By creating a set of circumstances to enable various types of impact to occur, these investments collectively represent a **purposeful **strategy intended to shape the actions of clinicians and health service managers within the health service” *[emphasis added].^3^ It is also clear that this was seen as a long journey towards achieving the intended impacts. It was, according to Brown and colleagues, *“a gradual, organic approach to developing research capacity with research becoming a key strategic pillar of the health service alongside clinical care and education.”*^2^ A whole-of-organisation focus was boosted in 2014 when it was adopted for the first time in a published THHS research strategy.

 What was achieved? The strategy made important progress, but faced considerable challenges: “*impacts were successful in isolated pockets, championed by individual researchers and facilitated by their policy and community-of-practice networks. However, there was little organisational-level support for continuity of research and implementation into practice and policy.”*^2^ The final sentences of the paper noted that further efforts would be needed if the ambition set out in the title was to be realised: *“Continuing investments should also involve actively supporting research translation and establishing ongoing, systematic processes for evaluating research investment and impact.”*^2^

## The Wider Context

 The THHS evaluation built on earlier work, including an evidence synthesis that explored whether improvements in patient care and health outcomes were associated with research-active clinicians and healthcare organisations.^4,5^ Perhaps the otherwise excellent and detailed THHS evaluation, could have unpacked a little further at the start how the impacts such as improvements in patient care and health outcomes were perceived in the evidence synthesis to which it referred?^4,5^ According to the synthesis, there were two main categories of impacts associated with research-active clinicians and healthcare organisations: Specific impacts and Broader impacts. Specific impacts arose as the clinicians and/or healthcare organisation in which research was conducted subsequently implemented the findings, if appropriate, to provide improved healthcare. This often occurred more rapidly than usual, because clinicians in the research organisation naturally tended know about, and perhaps trust, the findings from their own organisation more so than from others. Broader impacts arose as staff in a research-active healthcare organisation improved the healthcare provided because their research activities, and ideally the associated infrastructure, meant they were more willing and/or able to use research produced by others.^4,5^ Once these two types of impacts had been unpacked, then the mechanisms already carefully described in Brown and colleagues’ realist evaluation could have been applied to them.

 The 33 papers identified in this evidence synthesis were published before April 2012. They came from nine countries, with nearly half (15) from the United States and another five from Canada; 28 papers reported a positive association. What distinguishes these earlier papers from those evaluating the THHS approach is that almost two thirds of papers (21/33) focused on analysing the impact of research activities whose main purpose was to test new therapies or procedures and not, primarily, to seek general improvements in patient care at the research location through research engagement. When the latter impact was achieved, it was as categorised by the review team a by-product and was not the outcome of a purposeful strategy with that aim clearly articulated.

 Only a small percentage of the papers (4/33) were placed in an “intervention” group, which meant they described the impact of initiatives aimed at improving healthcare specifically at the research locations by involving healthcare staff in specific studies. But the aims of these four initiatives can also be distinguished from those at the THHS. It is a matter of scope. The four each focused narrowly on one medical field or problem such as rehabilitation for veterans or malnourishment in children: these were not intended to be wide-ranging whole-of-organisation initiatives.

 The remaining eight papers were in a middle category classified as network papers. All these came from the United States, reflecting what was, at that time, the more established nature of formal research networks in the United States.^4,5^ They were all positive.

 Therefore, a decade on from the data collection for the evidence synthesis, this THHS study illustrates there are attempts to develop research capacity primarily with the explicit aim of improving the healthcare provided across the board in the organisation in which the research is conducted, albeit with hopes for impact beyond the boundaries of the organisation as well. But can the challenges faced be fully answered at this organisational level? Or is it necessary to expand the focus beyond one hospital, and one regional health service (notwithstanding its large geographical area)?

## Further Efforts: Is a Systems Level Approach Needed?

 Arrangements in each nation and sub-national jurisdiction differ, but lessons can be learnt. In the United Kingdom the close alignment of research and healthcare was a key aim of a comprehensive research and development strategy that was launched in 2006, building on earlier initiatives.^6^ This strategy and its subsequent implementation helped to integrate research into the National Health Service (NHS), the UK’s universal healthcare system, through a range of system-wide research training and funding schemes and the development of research networks throughout the system. Additionally there were more local initiatives. Assessments of health outcomes of patients in NHS hospitals conducted after the evidence synthesis used data from the networks to compare health outcomes against levels of research activity.^7,8^

 Ozdemir and colleagues analysed data on risk-adjusted mortality and research activity in acute NHS trusts from 2010/2011 and their 2015 article concluded: *“Research active Trusts had lower risk-adjusted mortality for acute admissions, which persisted after adjustment for staffing and other structural factors.”*^7^

 Downing and colleagues focused on colorectal cancer and examined data for 2001/2008 from the cancer research network which was the first such research network established across hospitals in the NHS. In those early days not all trusts had such research activity. In their 2017 paper, they reported an association between the participation in trials and health outcomes using data from over 200 000 patients. Further, they identified that trusts with higher levels of research participation on average had better outcomes than those with some research participation, but at lower levels. The latter hospitals, in turn, did better than those without research participation. The authors concluded: *“There is a strong independent association between survival and participation in interventional clinical studies for all patients with CRC treated in the hospital study participants.... higher rates of participation and more years (of the eight studied) with high participation each showed a ‘dose effect.’”*^8^

 In these examples, networks helped to build research capacity and support activity across the healthcare system. The assumption was that the link between research participation and patient outcomes not only depended on the effective translation of the findings of studies conducted at the specific hospital but also reflected the benefits of having a broader research active environment.

 Furthermore, in 2018 the Care Quality Commission, which applies an inspection framework to assess the quality of care in each NHS trust, announced it was introducing additional questions into this framework. The new questions related to research activity, and included how well an NHS trust integrated research into its corporate strategy, as well as how well research opportunities were communicated to patients.^9^

 In January 2020, a report from the UK Academy of Medical Sciences noted that there was research activity in every NHS trust and referred to the evidence described above showing the association between research engagement and improved patient outcomes.^10^ But the report also claimed that there was evidence of a recent decline in the capacity of NHS staff to undertake or engage with research. Factors thought to contribute to this included a misalignment of incentives between the healthcare and academic systems, particularly in professions such as nursing and the Allied Health Professions where the percentage of staff classified as medical academics was extremely low.^10^

 In sum: adopting a systems approach in the UK strengthened research engagement and activity across the NHS, and associated improvements in healthcare outcomes have been demonstrated. But a key lesson is that challenges remain, challenges in some cases like those identified at THHS by Brown and colleagues.^2^ One challenge facing any attempts to achieve research impacts is the time it takes for impacts to arise. In some ways embedding research within a healthcare system can help. But achieving more rapid Broader impacts is likely to require some of the wider infrastructural investments around building research capacity and culture that the THHS evaluation identified as being insufficiently provided in most cases. As noted, the category called Specific impacts, ie, those arising from the implementation of successful locally conducted research, offer research active organisation an opportunity to accelerate impacts related to specific pieces of research. In the final sub-section we ask: how do the successes achieved in the pandemic offer support for embedding research systems into healthcare systems?

## Has COVID-19 Enhanced the Case for a Systems Approach?

 Data collection for the THHS evaluation ended in February 2020,^2^ as the COVID-19 pandemic began. As illustrated above, in the United Kingdom the pandemic dramatically highlighted the benefits of embedding a health research system in the healthcare system.

 Further insights are provided in two papers that somewhat mirror those from THHS by describing an equally rigorous detailed multi-method study of the experience of researchers in a single hospital organisation, again supported by illuminating quotes. However, this is a highly research-active UK NHS acute hospital trust, and the study considered how the hospital’s research workforce adapted to the pandemic as part of the system-wide National Institute of Health Research (NIHR) response.^11,12^ For the rapid success of the trials such as RECOVERY, the existing infrastructure of extensive research capacity built into the NHS was necessary, but not sufficient. Local ability to adapt was also crucial: “*the embedded research system was adapted and repurposed to support the COVID-19 response.”*^11^ Given their involvement in the RECOVERY trial, and the catastrophic outcome for many patients with COVID-19, UK hospitals were ready immediately to adopt the findings about the use of dexamethasone, and the extraordinarily high impact in terms of number of deaths averted was soon estimated.^1,13^

 The successes of the COVID-19 research responses from the NIHR embedded into the UK’s NHS were widely praised elsewhere. There were calls from leading researchers in Australia, Canada, the United States and across Europe for their research systems to become more like the NIHR with its integration into the healthcare system.^13^ In Australia, leaders of the AustralaSian COVID-19 Trial (ASCOT) found recruitment to their trial was much slower than to the RECOVERY trial.^14^ This was for a variety of reasons, including the much greater success of policymakers in Australia compared to the UK in using evidence-informed policies to control COVID-19 cases in 2020 and 2021. This meant there were fewer cases available for potential recruitment into trials in Australian hospitals.^13^ But Bowen and colleagues also noticed that in the research system network embedded across the UK’s health system “*RECOVERY was prioritised by the UK National Health Service as the preferred clinical trial in all its hospitals.”*^14^ They called for a similar system of prioritisation in Australia during a pandemic.

 In a report in November 2021, the Association of Australian Medical Institutes went further.^15^ While they claimed that the strong response across the board in Australia to the pandemic had been made possible by decades of investment building up health research capacity, they also noted some significant long-term problems that had been exposed by the pandemic. They stated: *“Better integration of research and healthcare, along with co-investment from state and federal governments, will enable the delivery of better patient outcomes.”*^15^

 Perhaps the pandemic, and the consequent calls for action from groups such as the Association of Australian Medical Institutes, will boost the momentum for a system-wide approach to integrating research into the Australian healthcare system? In the United Kingdom, members of the RECOVERY team have called for their success to be further built on through even greater integration of research into the UK healthcare system. This Commentary started by noting the title used by Brown and colleagues, perhaps inspiration could be drawn from the equally challenging title of the paper from the RECOVERY team: *“Making trials part of good clinical care: lessons from the RECOVERY trial.”*^1^ Both papers illustrate the importance of continuing to gather evidence to support the development of research systems: to that end, we are currently updating our original evidence synthesis.^4,5^

## Acknowledgements

 I am grateful to Bryony Soper for very helpful comments and suggestions on an earlier draft. Responsibility for the final version lies with the author.

## Ethical issues

 Not applicable.

## Competing interests

 Author declares that he has no competing interests.
